# A novel taping therapy for pain after arthroscopic shoulder surgery: study protocol for a randomised controlled pilot trial

**DOI:** 10.1186/s13063-018-2866-2

**Published:** 2018-09-24

**Authors:** Sun-Young Moon, Jung-Eun Kim, O-Jin Kwon, Ae-Ran Kim, Da-Heui Kim, Jae-Hong Kim, Hwa Soo Hwang, Changsop Yang

**Affiliations:** 1 Clinical Research Division, Korea Institute of Oriental Medicine, 1672 Yuseongdae-ro, Yuseong-gu, Daejeon, Republic of Korea; 2 Korean Medicine Life Science, University of Science and Technology (UST), Campus of Korea Institute of Oriental Medicine, Daejeon, 34054 Republic of Korea; 3 Department of Acupuncture & Moxibustion, DongShin University Gwangju Oriental Hospital, Gwangju, Republic of Korea; 4 Chims-Saengvit Oriental Medicine Clinic, Seoul, Republic of Korea

**Keywords:** Novel taping therapy, Interferential current therapy, Postoperative pain, Arthroscopic shoulder surgery, Randomised controlled trial

## Abstract

**Background:**

In recent years, the number of arthroscopic shoulder surgeries has increased given that the intervention is minimally invasive. However, postoperative pain is one of the most common symptoms of patients who undergo arthroscopic surgery. Although pharmacological treatments and brachial plexus blocks for reducing pain are currently used, the adherence rate of interventions is low, and adverse effects often occur. Chimsband, made up of silver and optic fibres, is a novel taping therapy that stimulates patients’ acupoints and is expected to relieve pain with few adverse effects. The aim of this study is to explore the effectiveness of Chimsband to relieve pain following arthroscopic shoulder surgery.

**Methods/design:**

This is a randomised, parallel, controlled, exploratory clinical trial. Thirty participants who undergo arthroscopic shoulder surgery will be randomly allocated to an intervention or a control group. Both groups will receive 10 sessions of interferential current therapy within a period of 2 weeks, while the intervention group will additionally receive taping therapy after undergoing physical therapy. Two follow-up visits will be scheduled after the last treatment session. The primary outcome variable will be the difference in the visual analogue scale (VAS) scores between baseline and first follow-up evaluation after the end of 10 treatment sessions. The secondary outcomes will be VAS at the end of the second week, shoulder pain and disability index, range of motion, VAS while sleeping, questionnaire of blood stasis pattern identification at two follow-up visits, and number of bands used per visit. Outcomes will be evaluated at baseline, 2 weeks from visit 1 (+ within 6 days) after commencement, and at 4 weeks from visit 1 (+ within 6 days) follow-up.

**Discussion:**

This study will be the first clinical trial to explore the effect and safety of Chimsband on postoperative shoulder pain. It would provide clinical evidence to conduct further taping therapy studies for relieving musculoskeletal pain.

**Trial registration:**

Korean Clinical Trial Registry, KCT0002355. Registered on 13 June 2017.

**Electronic supplementary material:**

The online version of this article (10.1186/s13063-018-2866-2) contains supplementary material, which is available to authorized users.

## Background

Shoulder arthroscopy is a minimally invasive surgical technique to treat shoulder pathology in the shoulder area [1]. Recently, there has been a preference by patients with shoulder injuries for arthroscopic surgery because of the short inpatient period and fast postoperative recovery [2]. The number of arthroscopic shoulder surgeries has increased by 600% from 1996 to 2006, while that of open rotator cuff repairs has increased only by 34% in the United States [3]. In Korea, over a period of 5 years (from 2010 to 2014), the number of patients with shoulder disease increased by an average of 4.6%, and medical expenses increased by an average of 13.3% annually. The number of shoulder surgeries (acromioplasty and ruptured rotator cuff repairs) reached 56,000 cases by 2014 [4] (http://www.medipana.com/news/news_viewer.asp?NewsNum=165805&MainKind=A, http://www.akomnews.com/?p=330302).

Patients who underwent arthroscopic shoulder joint surgery tended to experience more postoperative pain with delayed rehabilitation and increased dissatisfaction with surgical results compared to patients who underwent other arthroscopic surgeries [5, 6]. Pharmaceutical treatments are currently used to relieve postoperative pain. However, NSAIDs and long-term narcotic analgesics can induce nausea, vomiting and risk of addiction. A persistent brachial plexus block technique can be utilised in limited cases, as the risk of infection can increase. New treatment technologies such as non-pharmacological therapies and rehabilitation treatments with strengthening muscles that have fewer adverse effects (AEs) and are more effective are required [7].

There are several reports of complementary medicine that directly stimulate acupoints such as ST36, LI4 and ST6 as well as proximal points of painfulness area to control postoperative pain [8, 9]. Patients who underwent neck dissection and received acupuncture had increased Constant–Murley scores when evaluating pain and had improved shoulder function [10]. Electroacupuncture treatment also reduces postoperative thoracic pain [11]. Studies regarding indirect stimulation of acupoints, such as the PC6 (pericardium-6: Neiguan) point stimulation band, made up of a wrist band and a bead to stimulate the acupoint, which is effective for postoperative nausea, have been published as study protocols [12], while the effectiveness of indirect acupoint-stimulating therapies to relieve postoperative pain is still unknown.

**Fig. 1:**
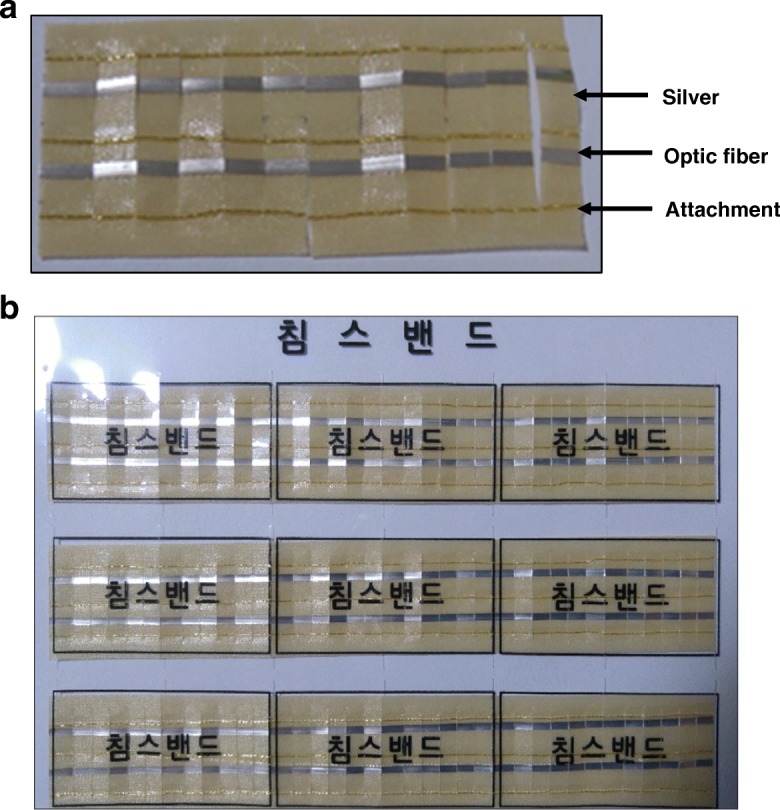
Appearance of Chimsband. **a** Individual Chimsband. **b** One set of Chimsband

Chimsband (Chimsband; Chims-Saengvit Oriental Medicine Clinic, Seoul, Korea) is a taping therapy invented by a Korean medical doctor, and it is also the name of a medical device. Chimsband comprises thin silver layers and optic fibres with an adhesive patch, with high electric and photic conductivity (Fig. 1). Painful areas have deviant bioelectric currents, and the fibres in Chimsband are supposed to recover abnormal areas on a patient’s body by equalising the biocurrent between normal area and painful sites. Although there are some case studies of Chimsband application for headache, paresthesia, chronic pain, diarrhoea, rhinitis, and depression, no randomised clinical trials to evaluate its clinical effectiveness have been conducted [13, 14].

This study is being conducted in order to explore the effectiveness and safety of Chimsband to control postoperative pain. The participants will be patients who have undergone arthroscopic shoulder surgery who have postoperative pain. This study will generate basic evidence on the effect of Chimsband and direct the topics of further studies. We hypothesise that treatment with Chimsband in addition to interferential current therapy (ICT) will be more effective than ICT treatment alone. We will test this hypothesis by a randomised controlled pilot trial. This study protocol was written in accordance with the Standard Protocol Items: Recommendations for Interventional Trials (SPIRIT). The SPIRIT checklist has been included in Additional file 1.

## Methods

### Design

This is a randomised, controlled, parallel-designed, exploratory clinical trial. It is an open-label study that will be conducted at DongShin University Gwangju Oriental Hospital, Korea.

**Fig. 2:**
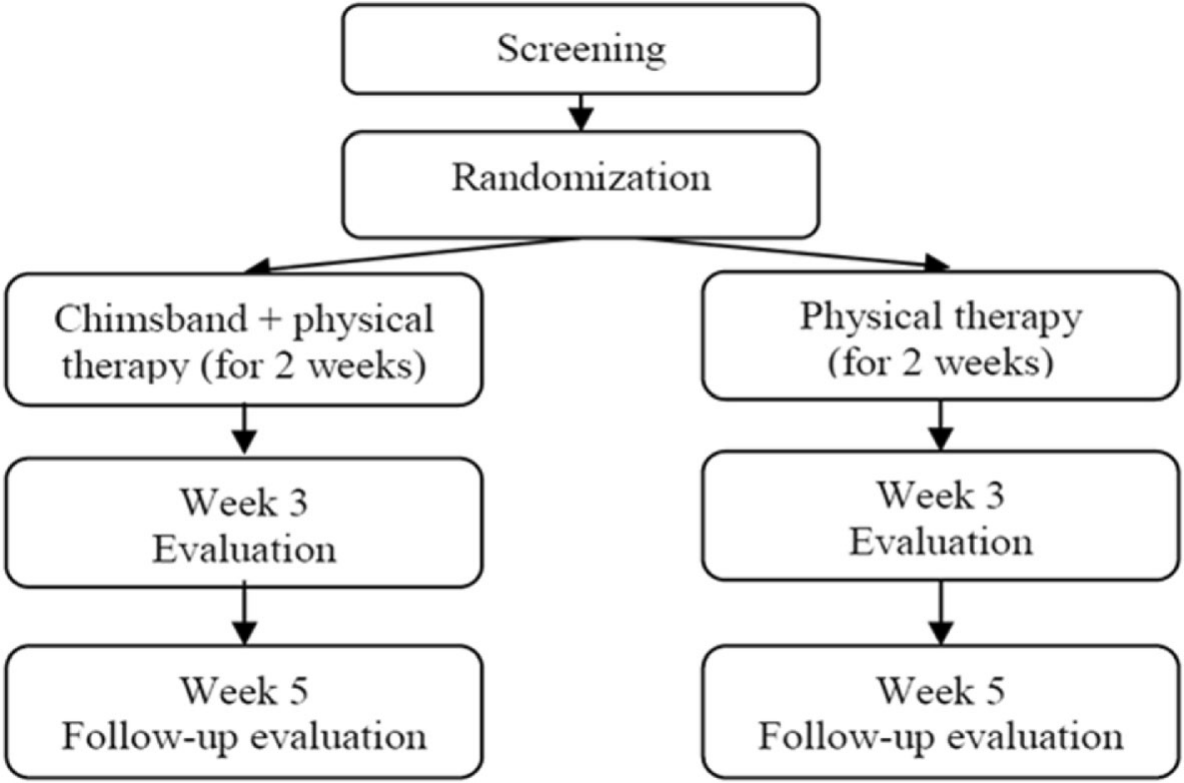
Flowchart of the clinical trial

Thirty participants who voluntarily submit written informed consent will be randomly allocated to either the intervention group or the control group if the participant is deemed eligible to enter the clinical trial. Both groups of participants will receive ICT therapy because of ethical issues. The intervention group participants will additionally receive 10 sessions of Chimsband therapy over a period of 2 weeks (+ 6-day window), while the control group participants will not receive band therapy. After intervention, both groups will be evaluated at week 4 from visit 1 (+ 6-day window), and the clinical trial will end after the follow-up assessment on each variable at week 4 from visit 1(Fig. 2).

### Inclusion criteria

Patients with postoperative pain after arthroscopic shoulder surgery who meet the following criteria will be included:

1. Between 19 and 69 years old (either sex)
2. Pain in the shoulder area after arthroscopic shoulder surgery
3. Underwent surgery within the past 2 years and at least 2 weeks previously (whose surgical fixation area has completely healed)
4. Visual analogue scale (VAS) score for pain is greater than 40 on initial screening
5. Voluntarily participated in the trial and submitted a written informed consent form

### Exclusion criteria

Patients meeting any of the following criteria will be excluded:

1. Patients who do not submit documentation that can verify arthroscopic shoulder surgery history (surgery admission letters, medical opinions, diagnosis form or medical certificates)
2. Patients who have contact dermatitis or scars surrounding the surgical sites and whose clinicians diagnosed them as inappropriate to enter the trial
3. Patients who have used narcotic analgesics upon initial screening
4. Patients with a clinical diagnosis of acute inflammation, including suppurations surrounding the shoulder surgery area and swelling or any laboratory test result that is more than 1.5 times that of the upper or lower limit of normal range during the screening
5. Enrolled in other clinical studies
6. Participants with drug or alcohol abuse history or suspicious of those
7. Accommodated in residential institutions such as social welfare institutions

### Randomisation and allocation concealment

A statistician not involved in the conduction and evaluation of the clinical trial will make the allocation list by block randomisation using Statistical Analysis System (SAS)^®^ Version 9.4 (SAS institute. Inc., Cary, NC, USA). The participants will be randomly assigned in a 1:1 ratio to the intervention group or control group. Participants who signed a written consent form will receive individual screening numbers. If the potential subject is eligible to participate in the clinical trial by screening, the statistician will allocate the randomisation numbers sequentially and finally determine the subject identification code. Once the number is allocated, reuse of the number is prohibited. If screened participants are not randomly allocated, the clinical research coordinator will record the individual numbers of participants not allocated and the reason patients were not allocated on the case report form.

Random allocation codes will be stored in opaque envelopes and kept in a double-locked cabinet. If the investigator decides to enrol a subject who signs informed consent and meets the inclusion/exclusion criteria, the investigator will open the randomised allocation envelopes in sequence with the participants and allocate subjects to each group. The investigator will record the opening date, sign the opened envelopes and store them separately.

### Interventions

The intervention group will receive ICT and additionally receive Chimsband therapy for 10 sessions over a period of 2 weeks. The control group will only receive ICT therapy. All intervention practitioners will not be involved in outcome assessment procedures.

#### Interferential current therapy (ICT)

The physical therapist will carry out ICT to all participants one time per visit (visit 1 to 10), taking into consideration patients’ shoulder and pain status. The ICT instrument will be the IN-2200 from the Youngin-Medical Corporation, the transportation frequency will be 3750 to 4000 Hz, and the interferential frequency will be 0 to 250 Hz, set to the automatically changed mode. A four-pad electrode will be arranged in a crisscross pattern on the patients’ area of pain. The current strength for therapy will be set at the level of patient tolerance. Strength will be printed in interrupt mode, and adaption time will be 20 min. For the treatment group, the physician will perform physical therapy after removing the Chimsband, and new Chimsband will be attached to the patient’s shoulder when the physical therapy ends.

#### Intervention group

**Fig. 3:**
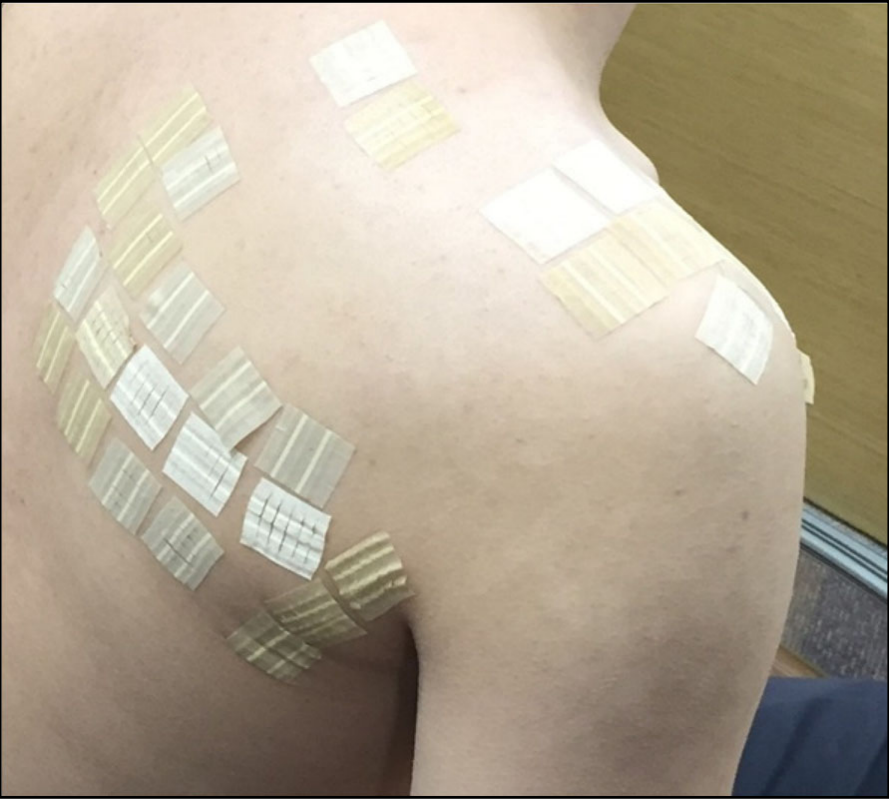
Intervention of Chimsband

There will be 10 treatment sessions over a period of 2 weeks (+ 6-window days), and physicians will attach Chimsband to patients at each visit. For the treatment group, Chimsband will be attached on the painful shoulder area, where acupoints LI15, LI16, GB21, SI10, and TE14, and Ashi points are located (Fig. 3). Physicians will define the Ashi points that produce a painful or uncomfortable sensation with pressure [15]. Only painful points located in the shoulder-dorsal area, cervicothoracic area and upper limb muscle area will be accepted. Patients will visit the physician at 2 and 4 weeks after visit 1 (+ 6-window days) to assess the effectiveness and safety of the treatment.

#### Control group

This trial proposes evaluating the overall effectiveness of Chimsband for postoperative pain. To reflect real-world treatment, the control group will be assigned to receive physical therapy. Physical therapists will carry out only ICT to the control group participants one time per visit. The treatment session will be 10 times over a period of 2 weeks (+ 6-window days). Patients will visit the physician at 2 and 4 weeks after visit 1 (+ 6-window days) to assess the effectiveness and safety of the treatment.

### Concomitant treatments

All participants in the intervention and control groups will be asked not to take other therapies intended to reduce postoperative pain from arthroscopic shoulder surgery other than the treatments in this clinical trial. If the participants have undergone continuous treatment before enrolling in the trial, there should be no changes in this treatment during the study period. All patients should report new treatments to the clinical trial institution when therapies are started after entering the clinical trial.

### Primary clinical outcomes

The primary outcome is shoulder pain and will be measured by the VAS measured at visit 11. If the score exceeds 40 points on screening, the participant will be enrolled in the study. Visit 11 will be at visit 1 + 2 weeks + 6-window days (last Chimsband attachment or ICT + 6-window days), as first follow-up date [16].

#### VAS on shoulder pain

Subjects will explain their pain severity on a scale. The left end of the line is 0 (no pain), and the right end is 100 (worst possible pain). Physicians measure the length from the zero point to the patient’s mark and convert it to the VAS score. Participants will indicate their previous night’s shoulder pain on the line by marking a horizontal line, and the assessor will measure the position of the recorded line [17].

### Secondary clinical outcomes

The secondary outcomes will be pain VAS at visit 12, Shoulder Pain and Disability Index (SPADI), range of motion (ROM), shoulder pain VAS while sleeping, blood stasis pattern identification questionnaire, and number of bands used per patients on each visit. All questionnaires will be presented in Korean.

#### Shoulder Pain and Disability Index (SPADI)

The SPADI questionnaire, assessing the pain and disability of shoulder joints, is composed of 5 items for shoulder pain and 8 items for shoulder disability to measure the shoulder mobility. Patients will check their discomfort by scoring each item from 0 to 10 [18, 19].

#### ROM of the shoulder

A goniometer (USA, iGaging, digital goniometer) will be used to measure the painful shoulders’ range of flexion, abduction, and internal and external rotation while the patients stand straight against the wall, and the sequence should be identical each time [20].

#### VAS of shoulder pain while sleeping

Patients will record the severity of their pain the previous night while sleeping by marking a vertical line on the VAS horizontal line.

#### Questionnaire based on blood stasis

Yang et al. [21] developed and verified the blood stasis questionnaire, including 14 articles (sprain, contusion, long-term numbness, tingling, pain in the lower belly, flank pain, nocturnal pain, feeling tumours in the abdomen, easily bruising, black face colour, blue or dark violet colour of the lips, tongue, gum, or eyelids, melena, and number of surgeries). Patients can express the strength of pain on a 7-point scale.

#### Number of bands used

Physicians will record the number of bands attached to each participant’s acupoints and the painful points of the shoulder on each visit.

### Other outcome measures

**Fig. 4:**
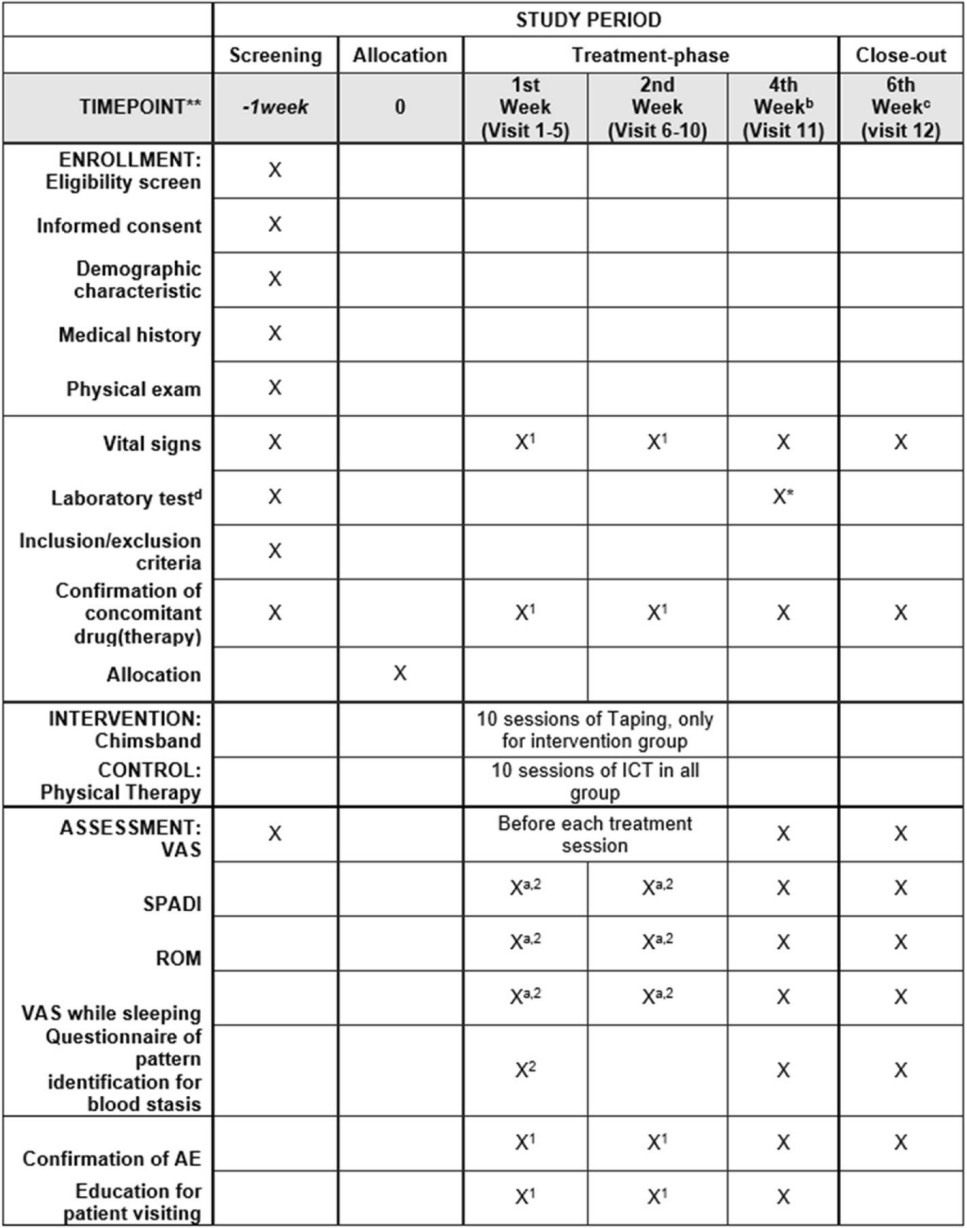
Schedule of enrolment, interventions and assessments

Feasibility outcomes of the study will include recruitment rate, completion rate and adherence to therapy. The ratio of included participants to all screened participants and the total enrolling target number will be presented as the recruitment rate. Therapy adherence will be shown as the percentage of each participant’s treatment frequency per total treatment frequency. Overall schedules of enrolment, interventions and assessments are shown in Fig. 4.

### Sample size

No prior research has studied the effectiveness of taping therapy on controlling pain from shoulder arthroscopy. According to a recommendation report on pilot studies in the bioscience field, if there is no prior article from which to calculate the sample size of the study, the size could be 12 per group [22]. Thus, for this study, the intervention and control groups need 12 participants. Therefore, 15 participants per group are needed, considering a 20% dropout rate.

### Statistical analysis

#### Primary analysis group

When analysing the effectiveness of clinical trials, the primary analysis group will be the full analysis set group, and the per protocol group will be additionally verified. For safety analysis, the intention-to-treat analysis group will be the primary analysis group, and the per protocol analysis group will be additionally verified.

#### General rule of data analysis

All statistical analyses in the trial will be two-sided sets, and *p* values will be 5%. SAS^®^ Version 9.4 will be used to analyse the data.

#### Primary clinical outcomes

Mean VAS variation difference between visit 1 and 11 will be tested by the following hypothesis.

Null-hypothesis (H0): There is no difference in the mean VAS variation between before and after treatment in each group.

Alternative hypothesis (H1): There is a difference between the mean VAS variation before and after treatment in each group.

Mixed-effect Model Repeated Measures (MMRM) will be adapted for fixed factors, namely treatment groups and visiting time, while random factors will be the participants in the trial.

#### Secondary clinical outcomes

VAS at visit 11, SPADI, ROM, VAS while sleeping, questionnaire of blood-stasis pattern identification, and the number of used bands on visits 11 and 12 (secondary follow-up, fourth week from visit 1 with + 6-day window) will be analysed by the same method as in the primary effectiveness evaluation. For analysing the difference of measurement value between before and after treatment in each group, Student paired *t* test or Wilcoxon signed-rank test will be adapted for effectiveness evaluation variables. To test the difference related to variations of VAS per visit, repeated measures analysis of variance will be conducted, and the adjustment method of multi-comparison will be Dunnett’s procedure. In addition, the subgroups will be analysed depending on the pain severity or post-operation stage.

#### Missing data

This study adopts the MMRM to analyse variations of evaluation parameters for the validity between groups. MMRM considers the missing values by maximum likelihood, not requiring any method of imputing missing values. Conversely, to reduce data loss, the missing values shall be substituted for the Last Observation Carried Forward method in case of adopting the Student paired *t* test (or Wilcoxon signed-rank test) to compare the pre- and post-treatment measurements in groups. Additionally, the Last Observation Carried Forward method shall replace the missing values in case if adopting the repeated measures ANOVA.

### Data handling and safety monitoring

Investigators will collect the data using standard operating procedures with Medidata Rave software (Medidata Solutions Inc., New York, NY, USA). The clinical research coordinator will record all AEs in the study period on the Case Report Form. The investigator will assess the AEs according to patient reports and interviews to verify adverse events, laboratory tests and vital signs. Every severe AE will be reported to the Institutional Review Board and sponsor following Good Clinical Practices and Ministry of Food and Drug Safety regulations. Data and safety reports will be regularly monitored to control the quality of the trial. A clinical research associate will check whether the trial followed the standard operating procedure, study protocol and enforcement rules with source data verification.

## Discussion

When non-invasive treatments fail to adequately treat patients with shoulder pain, shoulder arthroplasty is an essential and effective method to relieve pain and improve shoulder joint function. Minimally invasive arthroscopic shoulder surgery is beneficial for patients who desire less usage of analgesics and earlier return to work [23]. However, postoperative pain is often a concurrent problem that delays rehabilitation and lowers the quality of life [24]. Multimodal pain control, combining pharmaceutical and non-pharmaceutical techniques, is newly recommended to enhance recovery after surgery [25, 26]. Among these techniques, acupoint stimulations, such as acupuncture and electro-acupuncture, are reported as effective to reduce pain and the dosage of narcotics [8].

This study used the Chimsband as an acupoint stimulating method. This medical tape is a combination of adhesive patch, two thin silver lines, and an optic fibre. Chimsband was developed to normalise the bioelectrical current by attaching to the acupoints or on the Ashi points, which are painful areas that usually show abnormally high or low dermal biocurrents. Previous case studies reported their clinical effectiveness in reducing chronic pain and other symptoms from depressive disorders and sleep disorders [13, 16]. However, the mechanism behind the clinical results has not yet been proven in the experimental setting.

This is the first study to evaluate the effect and safety of Chimsband on patients with postoperative shoulder pain. It will utilise the VAS pain scale, SPADI, shoulder ROM, and questionnaires of blood stasis pattern identification as effectiveness parameters.

This clinical trial has several limitations, including its non-blinded design and small sample size; however, it has strength as a preliminary study to assess the potential effectiveness of Chimsband by a randomised controlled trial. Through this study, we expect to generate basic evidence of the effect of Chimsband on controlling pain. Further studies should be conducted with a larger sample size.

## Additional file

Additional file 1: SPIRIT 2013 Checklist: Recommended items to address in a clinical trial protocol and related documents. (DOC 123 kb)

## Abbreviations

AE: adverse effect
ICT: interferential current therapy
MMRM: Mixed-effect Model Repeated Measures
ROM: range of motion
SPADI: Shoulder Pain and Disability Index
VAS: visual analogue scale
